# Covalent functionalization of G protein-coupled receptors by small molecular probes

**DOI:** 10.1039/d4cb00294f

**Published:** 2025-02-14

**Authors:** Bert L. H. Beerkens, Adriaan P. IJzerman, Laura H. Heitman, Daan van der Es

**Affiliations:** a Division of Medicinal Chemistry, Leiden Academic Centre for Drug Research, Leiden University Einsteinweg 55 2333 CC Leiden The Netherlands d.van.der.es@lacdr.leidenuniv.nl; b Oncode Institute, Leiden The Netherlands

## Abstract

Roughly one-third of all marketed drugs act by binding to one or more of the >800 human GPCRs, primarily through activation or inhibition *via* the orthosteric binding site. In addition, novel strategies to alter GPCR functioning are being developed, including allosteric, biased and covalently binding ligands. Molecular probes play an important role in verifying such drug molecules with new modes of action and providing information on all factors involved in GPCR signalling. Various types of molecular probes have been developed, ranging from small molecules to antibodies, each bearing its own advantages and disadvantages. In this mini-review, a closer look is taken at small molecular probes that functionalize GPCRs in a covalent manner, such as through the conjugation of reporter groups like fluorophores or biotin. Covalently bound reporter groups allow the investigation of GPCRs across an increasing range of biochemical assay types, yielding new insights into GPCR signalling pathways. Here, a broad range of recently developed ‘functionalized covalent probes’ is summarized. Furthermore, the use of these probes in biochemical assays and their applications in the field of GPCR research are discussed. Lastly, a view on possible future applications of these types of small molecular probes is provided.

## Abstract 2

In this review, we take a closer look at the most recent small molecular probes that covalently label G protein-coupled receptors.

## Graphical abstract

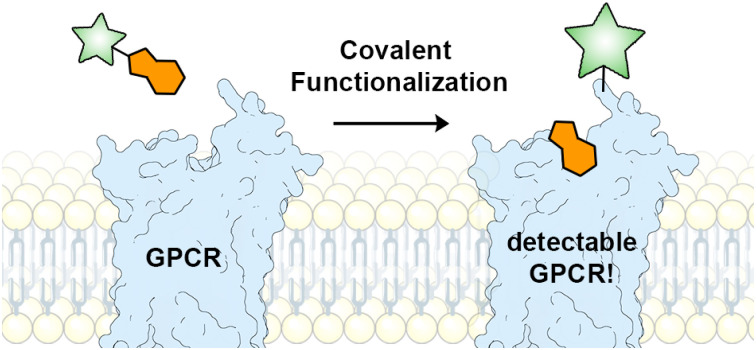

## 1. Introduction

G protein-coupled receptors (GPCRs) are transmembrane proteins that function as sensors, enabling cells to respond to molecules in the extracellular environment. Upon binding to extracellular stimuli, GPCRs undergo conformational changes, triggering a cascade of intracellular signalling events. Signalling pathways initiated by GPCRs can significantly influence cellular physiology, and many pathophysiological conditions have been linked to the activation or malfunction of GPCRs.^1^ Such findings have led to a surge in GPCR drug discovery at the end of the 20th century, resulting in over 500 currently marketed drugs targeting GPCRs.^2^ Besides the development of ‘classical’ orthosteric ligands, current strategies to modulate GPCR functioning include new types of small molecules, such as biased, allosteric, bitopic and covalent ligands.^3^ To take advantage of these novel modulation strategies, it is important to study these ligands and understand their molecular mechanisms of action. Moreover, new drug discovery efforts would greatly benefit from increased insights into GPCR signalling pathways in general. Fortunately, chemical and biological probes are being developed as tools to aid the molecular and pharmacological characterization of GPCRs.^4–7^ The utilization of the right type of probe can help overcome certain limitations, such as the low expression levels of GPCRs, and facilitate the study of GPCR pathways involved in the conditions of interest.

Historically, radioactive chemical probes have been a primary resource for GPCR characterization.^8^ β- and γ-emitting radioligands are used to precisely determine the binding affinity of putative ligands, while positron-emitting radioligands are being used to trace GPCR distribution *in vivo*. Radiolabelled chemical probes, however, require the use of radioactive material, specialized labs and additional waste treatment. Therefore, fluorescent ligands have emerged as alternative chemical probes for GPCRs.^6,7^ Fluorescent ligands are useful molecular tools in compound screening and aid in determining the subcellular localization and cellular expression levels of GPCRs, in combination with fluorescent plate readers, confocal microscopes and flow cytometers. Additionally, GPCR-targeting antibodies are being developed as biological probes. The use of GPCR antibodies, however, is not without challenges.^6^ While some antibodies have shown successful applications,^9,10^ other antibodies suffer from low selectivity towards their target GPCR.^10–12^ One reason for this lack of selectivity is the low number of possible unique epitopes: the extracellular portion of a GPCR that functions as an antibody recognition site ‘merely’ comprises an N-terminus and three extracellular loops. The length of these extracellular domains, and thus the ability to be selectively targeted by an antibody, differs greatly for each GPCR.

Altogether, there is a broad overlap between the applications of radioligands, fluorescent ligands, and antibodies. However, these chemical and biological probes all bind in a reversible fashion. A different strategy to study GPCRs is through covalent functionalization. Here, the GPCR is covalently functionalized with a detection group of interest, such as a fluorophore, biotin moiety, or ‘click’ handle. A big advantage of covalent functionalization is the robustness of the bond between the GPCR and the detection group, allowing the inclusion of washing steps, reductants, oxidants, surfactants, and other chemicals in biochemical assays. This allows the investigation of GPCRs by an expanded set of experimental methods, including SDS-PAGE and pull-down proteomics.

**Fig. 1:**
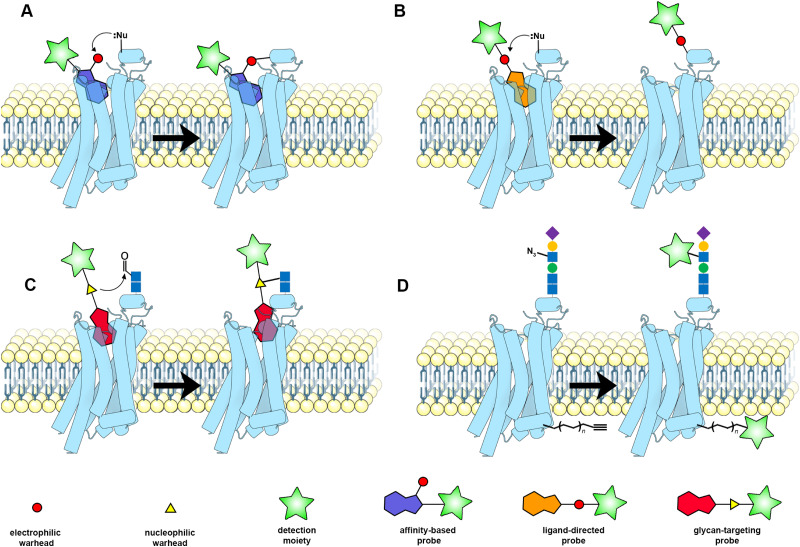
Schematic of the various functionalized covalent probes described in this review. (A) Affinity-based probes (AfBPs). After reversible binding of an AfBP to the target GPCR, covalent bond formation occurs between the warhead of the AfBP and a nearby amino acid residue, in this example, an electrophilic warhead and a nucleophilic amino acid residue, resulting in the irreversible conjugation of the probe and detection moiety to the GPCR. (B) Ligand-directed probes. Upon reversible binding of a ligand-directed probe to the target GPCR, a nearby nucleophilic residue attacks the electrophilic warhead, resulting in intramolecular bond cleavage and subsequent donation of the reporter group to the GPCR. (C) Glycan-targeting probes. First, aldehydes are generated through chemically induced oxidation of the extracellular glycan chain of the GPCR. Next, the glycan-targeting probe binds reversibly to the GPCR, and the nucleophilic warhead attacks the generated aldehyde, resulting in covalently bound glycan-targeting probe. (D) Metabolically incorporated probes. First, fatty acids or sugar molecules derivatized with click groups are added to the cell culture medium. These molecules are then post-translationally incorporated into the GPCR structure, allowing functionalization of the GPCR *via* click chemistry. This figure was partly generated with Protein Imager,^13^ using the structure of the adenosine A_2A_ receptor (PDB: 7ARO).

In this mini-review, we discuss the recently reported small molecular probes that are able to covalently functionalize GPCRs. As the term ‘covalent probes’ is already used to describe covalent ligands, we use the term ‘functionalized covalent probes’ or ‘functionalized covalent ligands’ throughout this mini-review. As such, we hope to emphasize both the reactive groups and the reporter groups. Although covalent GPCR functionalization might also be done through genetic alterations,^5,6^ these strategies are not applicable to native GPCRs and are therefore beyond the scope of this review. Here, we discuss most, if not all, of the recently developed small molecular probes that have been used to covalently functionalize GPCRs and briefly highlight their interesting applications. Four different types of functionalized covalent probes are discussed: affinity-based probes (AfBPs) (Fig. 1(A)), ligand-directed probes (Fig. 1(B)), glycan-targeting probes (Fig. 1(C)), and metabolically incorporated probes (Fig. 1(D)), with each labelling GPCRs in their own specific manner.

## 2. Affinity-based probes

AfBPs are tool compounds that consist of three functional moieties: (1) a high-affinity ligand that promotes selective binding to the protein target of interest, hence the term ‘affinity-based’; (2) a reactive group (‘warhead’) that induces covalent binding to the protein target; (3) a reporter group that allows detection of the probe-bound protein in biochemical assays (Fig. 1(A)). Although such probes have been synthesized for GPCRs for over three decades, the term ‘affinity-based probes’ is a recent derivation of the term ‘activity-based probes’ that was first coined by Cravatt and co-workers.^14^ While activity-based probes are similar tool compounds, they differ in reactivity, as their warheads target nucleophilic amino acid residues within the active site of enzymes. GPCRs on the other hand, do not have such an active site nucleophile that can be targeted. Therefore, AfBPs for GPCRs require relatively more reactive warheads.

Two types of warheads can be distinguished: photoreactive groups and electrophilic groups. AfBPs with photoreactive groups, also named ‘photo-affinity probes’, covalently bind their target GPCR upon irradiation at specific wavelengths. Dependent on the type of photoreactive group, carbene or nitrene species are generated, which will then insert into neighbouring hydrogen–heteroatom bonds.^15^ Due to this broad reactivity, most photo-affinity probes do not require a particularly reactive amino acid residue to be present in the binding pocket of the receptor. However, their broad reactivity might also cause an increased amount of off-target labelling. Electrophilic AfBPs on the other hand, covalently bind their target GPCR through the attack of a proximate nucleophilic amino acid residue. Here, specific labelling of the target GPCR requires a balanced electrophile, *i.e.* one that is reactive enough to be attacked by the weakly nucleophilic amino acid in the ligand binding pocket, but will be not randomly attacked by any amino acid residue in the proteome.^16^

Considering the third functional moiety of AfBPs, the reporter group, a distinction can be made between ‘one-step’ and ‘two-step’ probes.^17–19^ In the case of one-step AfBPs, the reporter group, *e.g.* a fluorophore or biotin moiety, is directly conjugated to the probe. Two-step AfBPs, on the other hand, contain a bio-orthogonal group (click handle) that can be functionalized after covalent binding to the target GPCR. Multiple probes have been developed that contain either an alkyne, azide, or *trans*-cyclooctene group that can be functionalized using click chemistry. The advantage of two-step AfBPs is the lack of bulky reporter groups, which might otherwise strongly influence the affinity towards the GPCR of interest. The disadvantages of two-step AfBPs are the introduction of an extra ‘click’ step in the assay protocol and the possible use of reagents that could disrupt the cells. In the next paragraphs, the most recent advancements in the development of photo-affinity and electrophilic AfBPs for GPCRs are discussed.

### 2.1. Photo-affinity probes

The introduction of photoreactive groups in the molecular structure of GPCR ligands has a long history, as photo-affinity ligands have been widely and long used to decipher the location of binding pockets in GPCRs.^20^ In some cases, photo-affinity ligands have been equipped with reporter groups, such as a fluorophore, biotin, or a radioisotope, to allow detection of the probe-bound residues by SDS-PAGE and mass spectrometry. More recently, technological advances in the fields of microscopy and mass spectrometry have led to new endeavours to use photo-affinity ligands in studies of GPCRs. Therefore, we aimed to take a closer look at the usage, as well as some exemplary applications, of GPCR-targeting photo-affinity probes from the past ∼10 years.

Over the past decade, photo-affinity probes have been developed for a multitude of GPCRs, most often targeting receptors that are interesting from a drug-discovery perspective. Some examples include probes for neurological receptors, such as the cannabinoid receptors,^21,22^ dopamine receptors,^23–25^ metabotropic glutamate receptors,^26,27^ opioid receptors,^28,29^ and serotonin receptors,^30^ and also probes for the calcium sensing receptor (CaSR),^31^ formyl peptide receptor 1 (FPR1),^32^ GPR39,^33^ GPR75,^34^ GPRC5A,^35^ and neurokinin 1 receptor (NK_1_R).^36^ All of these photo-affinity probes were developed based on endogenous molecules or known ligands, either with or without prior knowledge of their binding mode towards the respective receptor target. They were functionalized by conjugation towards a biotin moiety for pull-down (‘receptor capture’) experiments,^23,24,30,33,36^ a tetramethylrhodamine (TAMRA) fluorophore for flow cytometry and imaging,^32^ or a click handle to conjugate either biotin or a fluorophore to the probe-bound receptor.^21,22,25–29,31,33–35^

**Fig. 2:**
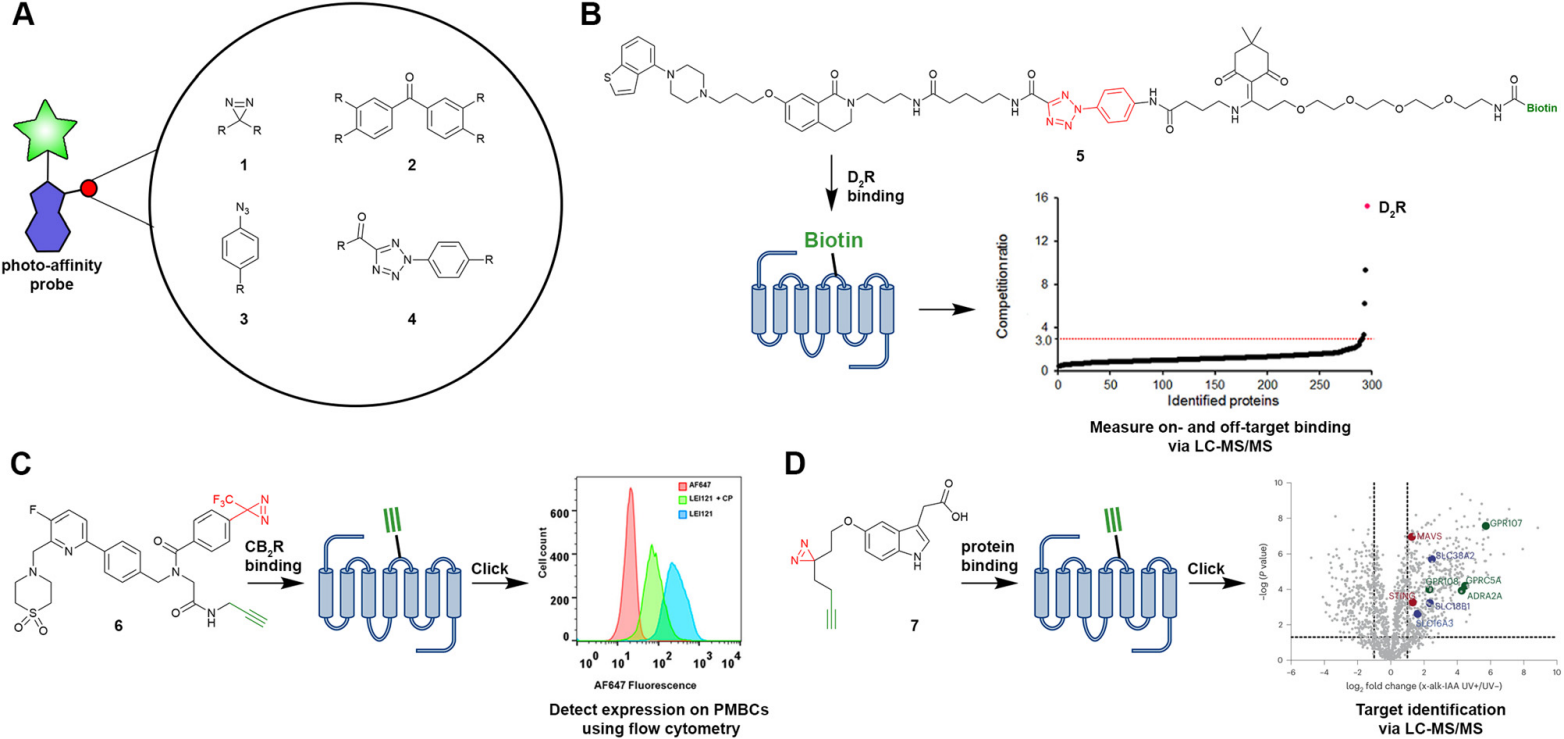
Selected photo-affinity probes, their warheads and exemplary applications. (A) Photoreactive groups that have been implemented in photo-affinity probes for GPCRs. (B) Probe 5 was used to measure on-target binding towards the D_2_R, as well as off-target binding of the respective piperazine benzothiophene scaffold.^24^ (C) Compound 6 (LEI121) was used to measure CB_2_R expression on PBMCs.^21^ (D) Indole metabolite-derived probe 7 (x-alk-TA) was used for target identification studies.^35^ Warheads are shown in red, reporter groups and click handles are shown in green.

Most of the recently reported photo-affinity probes contain a diazirine moiety (1) as the photoreactive group. One reason for the popularity of the diazirine group might be its improved synthetic accessibility.^15^ Moreover, the diazirine group is often chosen due to its small size, whereby it will cause minimal perturbance within the binding site of the GPCR of interest. Other photoreactive groups include benzophenone (2) and phenyl azide (3) moieties (Fig. 2(A)); however, due to their size, they require more rational implementation into the scaffold of photo-affinity probes. In a comparison study, Miyajima *et al.* synthesized various photo-affinity probes for the dopamine D2 receptor (D_2_R), containing either of the above-mentioned photoreactive groups, as well as the photoreactive 2-aryl-5-carboxytetrazole (ACT) group (4).^24^ Most interestingly, they found that the ACT-containing probe 5 (Fig. 2(B)) would bind to fewer off-target sites in proteomic pull-down experiments. Such findings highlight that not only the ligand design, but also the off-target reactivity are important considerations when designing GPCR-targeting photo-affinity probes.

Evaluation of binding towards the respective target GPCR has mostly been carried out to date by radioligand displacement experiments or functional assays, while labelling of the target GPCR has been evaluated by SDS-PAGE, flow cytometry, microscopy, or proteomic pull-down experiments. Most of the prior studies have focussed on the development of photo-affinity probes *per se* and therefore, understandably, not touched upon the further uses of the respective probes. Nevertheless, there are some notable examples of applications of photo-affinity probes, as discussed below.

First of all, an interesting application of photo-affinity probes is the detection of the relative expression levels of the particular target receptor among different cell types. For example, Soethoudt *et al.* transformed a selective agonist for the type 2 cannabinoid receptor (CB_2_R) into the clickable photo-affinity probe 6 (LEI121) (Fig. 2(C)) and used this probe to label CB_2_R on human peripheral blood mononuclear cells (PBMCs).^21^ Upon analysing the probe-bound cells using flow cytometry, it was found that CB_2_R expression could be compared between different types of immune cells, of which the highest expression was found on CD19^+^ B cells. Interestingly, the results on the protein level corresponded to the relative amounts of CB_2_R found on the mRNA level, as determined by qPCR experiments.

A second interesting application of photo-affinity probes is their use as a tool for the target identification of endogenous molecules,^34,35,37^ and hits from phenotypic screenings,^29^ that act *via* an unknown mechanism of action. An exemplary study is the recent work by Zhao *et al.*, who transformed the metabolite indole-3-acetic acid into the clickable photo-affinity probe 7 (x-alk-IAA) (Fig. 2(D)).^35^ Chemical proteomics experiments were then performed using 7 to pull down all the proteins targets of indole-3-acetic acid. While it was found that many different proteins were bound by 7, which was not surprising considering the relatively high concentration of the probe used (100 μM), the authors managed to pin down the orphan receptor GPRC5A as a target of various indole metabolites, as confirmed by multiple follow-up assays.

### 2.2. Electrophilic affinity-based probes

The current general interest in drugs and ligands bearing electrophilic groups is increasing,^16^ with a concomitant increase in the development of covalent ligands for GPCRs.^38^ While cysteine residues are the most frequently addressed targets of covalent ligands, mainly due to their favourable ‘soft’ reactivity at physiological pH,^16^ cysteine residues are relatively scarcer on GPCRs. Therefore, other amino acid residues have been targeted by covalent GPCR ligands, including lysine and tyrosine residues, requiring slightly more reactive electrophiles. Most of the published electrophilic AfBPs to date have been based on such previously developed covalent ligands. However, the development of electrophilic ligands and functionalized probes requires extensive fine-tuning; for instance, the warhead must be positioned at the right location, and must bear the right reactivity to allow covalent binding. Presumably due to these extra efforts required, the surge in interest and advancements in electrophilic AfBPs has been less steep than that for photo-affinity probes. Nevertheless, over the past decade, electrophilic AfBPs have been developed for adenosine receptors,^39–42^ the CC chemokine receptor type 2 (CCR2),^43^ glucagon-like peptide 1 receptor (GLP1R), and neurotensin receptor 1 (NTSR1).^44^

**Fig. 3:**
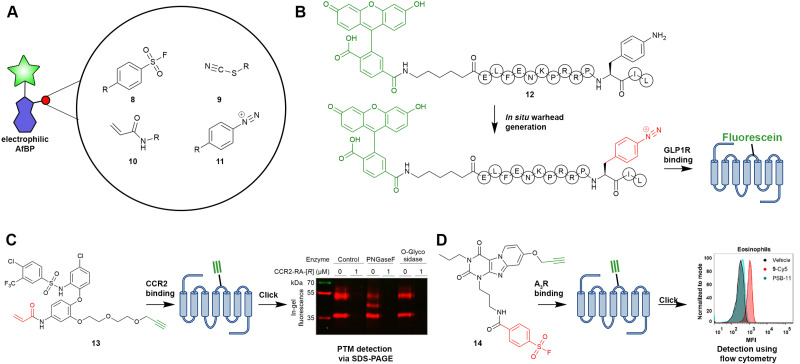
Selected electrophilic AfBPs, their warheads and exemplary applications. (A) Warheads implemented in the design of AfBPs. (B) Strategy for the *in situ* generation of aryl diazonium warheads, as shown for the GLP1R and NTSR1.^44^ (B) Probe 13 (LUF7834) was used for the detection of various CCR2 proteoforms.^43^ (D) Probe 14 (LUF7960) was used for the detection of A_3_R expression in human granulocytes.^42^ Warheads are shown in red, reporter groups and click handles are shown in green.

Adenosine receptor AfBPs all contain an aryl fluorosulfonyl group (8) as the warhead (Fig. 3(A)), mostly inspired by the electrophilic adenosine receptor ligands published over two decades ago.^45^ Our research group also found that aryl fluorosulfonyl groups have an appropriate reactivity, but only if used at low (<1 μM) concentrations.^41,42^ In the case of CCR2, cysteine residues are present in the intracellular ligand binding pocket, which allows for the implementation of more soft electrophiles in the design of AfBPs.^43^ During our initial efforts to develop CCR2 AfBPs focussed on thiocyanate-bearing AfBPs (9), we noticed that CCR2 binding was hampered in SDS-PAGE and proteomic pull-down experiments, presumably due to the reversibility of the probe-CCR2 bond. Acrylamide-bearing AfBPs (10), on the other hand, showed rigid covalent binding to CCR2 and survived the assay conditions. Lastly, aryl diazonium (11) groups have recently been reported as electrophiles to target GLP1R and NTSR1 *via* a proximity-induced azo coupling with tyrosine or histidine residues.^44^

For designing AfBPs, Sharma *et al.* recently presented an interesting strategy for the rapid synthesis of peptide probes.^44^ In their work, the unnatural amino acid 4-aminophenylalanine was included into the sequence of the peptide ligand 12. Treatment of the synthesized peptide with sodium nitrite transformed the aniline into the above-mentioned diazonium ion, generating the desired electrophile *in situ* (Fig. 3(B)). The authors applied this strategy to develop probes for both GLP1R and NTSR1 and showed the successful labelling of the two receptors by either western blot or confocal microscopy. Such synthetic strategies might lower the barrier for the implementation of electrophilic warheads and the application of electrophilic AfBPs.

Also in the case of the electrophilic AfBPs, most studies have focussed on probe development *per se*, although some interesting examples of applications have been reported. For example, in our study of CCR2, we investigated post-translational modifications (PTMs) through SDS-PAGE. By incubation with various glycosidases, followed by subsequent CCR2 labelling by AfBP 13 (Fig. 3(C)), we found that PNGaseF caused a reduction in the molecular weight of CCR2, thereby indicating the presence of one or more N-linked glycan chains. While similar SDS-PAGE experiments were carried out as early as the 1980s, using radioligands or antibodies, current strategies using fluorescent AfBPs offer a more accessible approach towards studying GPCR targets of interest. Thus, even though the concept has been around for some time, we think that new AfBPs can aid studies towards (the role of) PTM on GPCRs.

Further applications of electrophilic AfBPs are also in line with those of photo-affinity probes, but without the need for an extra irradiation step. However, caution must be taken as off-target labelling might occur due to side reactions of the used warheads.^41^ Similar to the aforementioned study on CB_2_R, we utilized AfBP 14 (LUF7960) in flow cytometry experiments to specifically label A_3_R on human immune cells (Fig. 3(D)).^42^ Although we did not detect the presence of the A_3_R on the most prevalent PBMCs (in-house data), we observed specific A_3_R labelling on eosinophils, which was in line with previous literature. Especially for A_3_R, antibodies have been found to suffer from low selectivity, presumably due to the minimalistic extracellular domains of A_3_R.^10,42^ Thus, in the case of A_3_R, small molecular probes such as AfBPs might offer a solution to study relative expression levels.

### 2.3. Broad-spectrum affinity-based probes

**Fig. 4:**
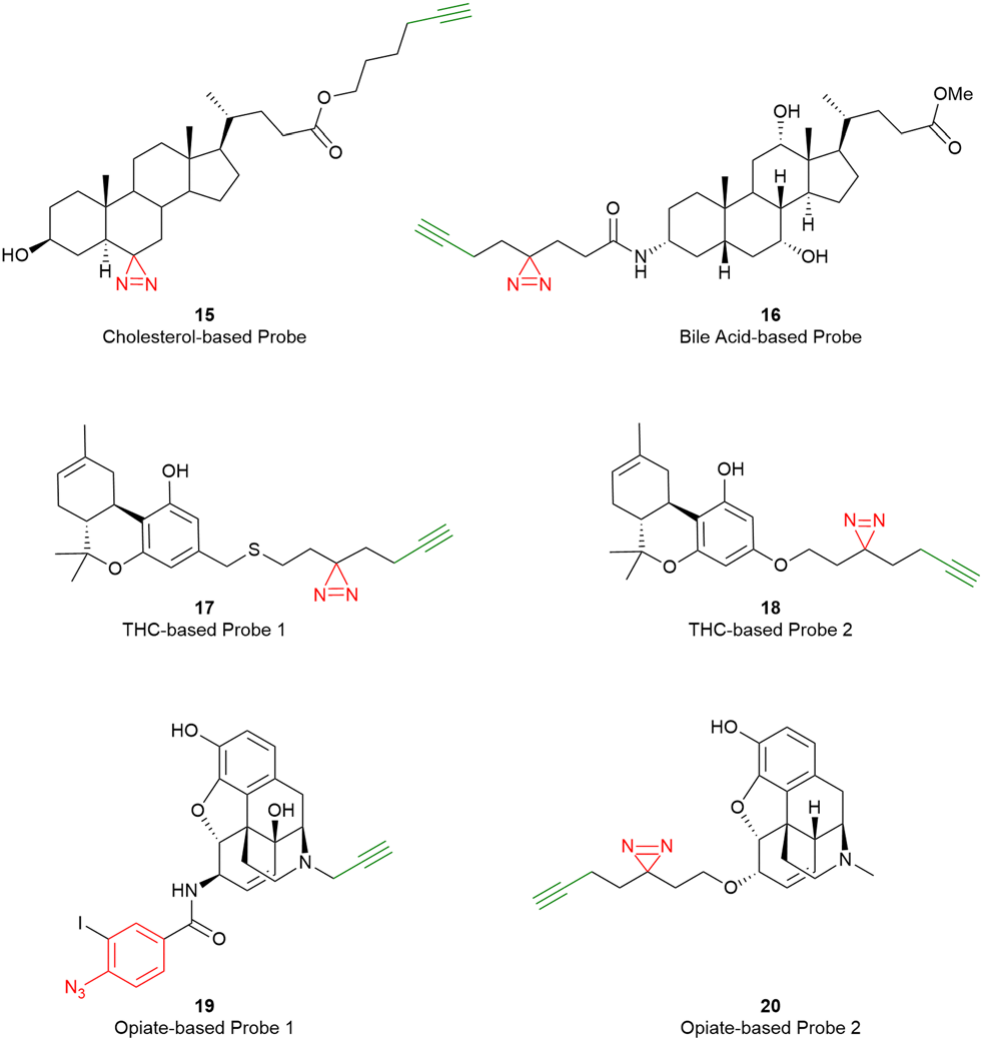
Exemplary AfBPs that might function as broad-spectrum probes.^28,29,49–52^ Photo-reactive warheads are shown in red, and alkyne groups for the implementation of reporter groups *via* click chemistry are shown in green.

Besides targeting one specific GPCR, a (sub) family of GPCRs might also be targeted by a ‘broad-spectrum’ AfBP. Broad-spectrum activity-based probes are already widely used in proteomic studies towards various families of enzymes, such as hydrolases, proteases, and kinases.^46–48^ In the case of GPCRs, the ‘high-affinity’ moiety of the probe should be a molecular scaffold that can bind to multiple GPCRs. Steroids are a good example, as these molecules have been shown to allosterically bind GPCRs (e.g. in crystal structures). In fact, both cholesterol and bile acid have been transformed into broad-spectrum photo-affinity probes 15 and 16 for proteomics studies.^49,50^ Also, Δ8/9-tetrahydrocannabinol (THC)-based probes 17 and 18 have been developed for investigating all the THC-binding proteins besides their target cannabinoid receptors, while 19 and 20 were also reported as opioid-derived photo-affinity probes (Fig. 4).^28,29,51,52^ However, the number of GPCRs detected by these broad-spectrum probes is still smaller than expected, presumably due to a multitude of factors, including the low expression levels of GPCRs compared to other targeted proteins, the lack of solubility of the membrane proteins in standard buffers, and the choice of digestion enzyme. For example, the C–X–C motif chemokine receptor 4 (CXCR4) is one of the GPCRs that was detected by probe 15, though it was presumably picked up due to its relatively high expression levels in the cell line used.^53^ Further enriching GPCRs, as well as decreasing the off-target reactivity towards other protein classes, is therefore necessary for the future detection of GPCRs using broad-spectrum AfBPs.

## 3. Ligand-directed probes

Ligand-directed probes are very similar to AfBPs as they also consist of three functional moieties: (1) a high-affinity ligand that induces selectivity, (2) an electrophilic group that reacts with a nucleophilic amino acid residue, and (3) a reporter group for detection in chemical biological assays. The main difference between AfBPs and ligand-directed probes is the electrophilic group. Upon reacting with a nucleophilic amino acid residue, the electrophilic group of a ligand-directed probe induces bond cleavage between the high-affinity ligand and the reporter group (Fig. 1(B)), allowing the high-affinity ligand to leave the binding pocket after donation of the reporter group to the protein. Ligand-directed probes are therefore attracting interest as new tools to label native GPCRs, without occupying the GPCR ligand binding pockets, allowing the investigation of *e.g.* agonist-induced signalling.

The idea of ligand-directed probes was developed by Hamachi and co-workers, who investigated multiple electrophilic groups for use in ligand-directed chemistry, including tosyl, dibromo benzoate, acyl imidazole, and *N*-acyl, *N*-alkyl sulfonamide (NASA) groups.^54–57^ The same team also provided evidence of the first ligand-directed probe capable of tagging a GPCR.^55,58^ In the past decade following this seminal work, multiple research groups have followed suite, resulting in a recent surge in the study and development of ligand-directed probes as tools to study GPCRs. To date, ligand-directed probes have been reported for the adenosine receptors,^59–62^ bradykinin receptor B_2_ (B_2_R),^58,63^ CB_2_R,^64^ dopamine D1 receptor (D_1_R),^65^ metabotropic glutamate receptor 1 (mGluR1),^66^ opioid receptors,^67,68^ and smoothened receptor (SMOR).^69^

**Fig. 5:**
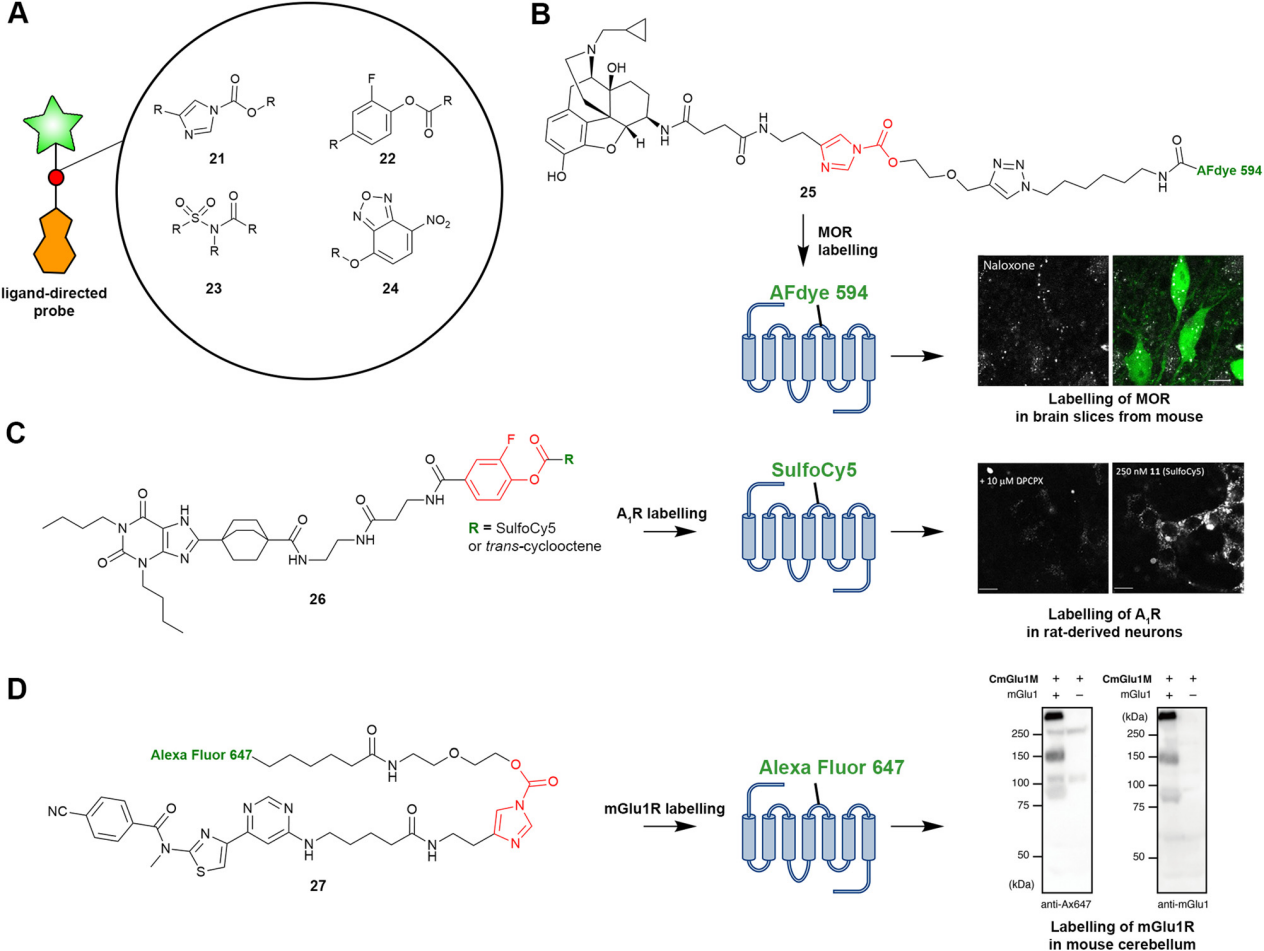
Selected ligand-directed probes, their warheads and exemplary applications. (A) Warheads implemented in the design of LD probes. (B-D) LD probes used for ligand-directed labelling of their respective target GPCRs in mouse- and rat-derived neurons or brain slices. (B) LD probe 25 (NAI-A594) was used to visualize MOR in brain slices from rats.^67^ (C) LD probe 26 was used to visualize the A_1_R on rat-derived neurons.^62^ (D) LD probe 27 (CmGlu1M) was used to visualize the mGlu1R in mouse cerebellum.^66^ Warheads are shown in red and reporter groups are shown in green.

The utilization of the acyl imidazole group (21) as an electrophile for the ligand-directed labelling of GPCRs was reported to be a successful strategy, and ligand-directed acyl imidazole (LDAI) probes have been applied to label various target GPCRs (Fig. 5(A)).^66,67^ Additionally, 2-fluorophenyl esters (22) have been used as electrophiles for the ligand-directed labelling of the A_1_R and the adenosine A_2A_ (A_2A_R) receptors.^60,62^ Interestingly, A_2A_R probes bearing a 2-nitrophenyl ester appeared to irreversibly block the orthosteric binding pocket, which was not the case for 2-fluorophenyl esters.^59,60^ However, these different observations might also have been caused by the different reporter groups used, which might or might not have occupied the ligand binding pocket, dependent on their size and structure.

Our group was particularly interested in the use of the NASA group (23) as a warhead for the ligand-directed labelling of the adenosine A_2B_ receptor (A_2B_R).^61^ Our interest came from the idea to rapidly convert the fluorosulfonyl group of our covalent ligands into the desired sulfonamide. However, we found our chosen NASA group to be too reactive in our experiments, resulting in a low signal-to-noise ratio and a lack of a specific GPCR signal in the performed biochemical assays. Building on the development of NASA warheads, Hamachi and co-workers reported the second generation of NASA warheads with a reduced intrinsic reactivity.^70^ Such warheads might offer a solution when targeting lowly abundant GPCRs. Lastly, an interesting ligand-directed labelling strategy was the use of an *O*-nitrobenzoxadiazole group (24) as a both electrophile and fluorophore.^64,69^ Upon nucleophilic attack by a proximal lysine residue, the moiety becomes fluorescent, resulting in a measurable ‘turn-on’ signal upon receptor binding.

While there are multiple examples of biotin and click tags as reporter groups for LD probes, most LD probes to date have been conjugated to a fluorophore for detection in biochemical assays. This has allowed the labelling of target GPCRs on live cells, mouse- and rat-derived neurons, and even *in vivo*, measured by subsequent analysis through microscopy or flow cytometry experiments.^62,64–67^ Arttamangkul *et al.* reported the first use of an LD probe to label GPCRs on brain slices.^67^ In their work, the opioid receptor antagonist naltrexamine was conjugated to an acyl imidazole electrophile, which in turn was connected to an Alexa 594 fluorophore (Fig. 5(B)). While their LD probe 25 showed affinity for multiple subtypes of opioid receptors, fluorophore labelling could be prevented by pre-incubation with a selective μ opioid receptor (MOR) antagonist, indicating the selective labelling of the MOR on the studied brain slices. This work paved the way for other studies to utilize LD probes to visualize GPCRs on neuron-derived cells.

For example, Comeo *et al.* reported the development of LD probe 26 to selectively label A_1_R.^62^ The probe design was based on the xanthine structure, which is well known for antagonizing adenosine receptors, and conjugated to a 2-fluorophenyl ester connected to either a SulfoCy5 fluorophore or a *trans*-cyclooctene group for click chemistry (Fig. 5(C)). The fluorescent labelling was blocked upon pre-incubation with an A_1_R antagonist, indicating selective labelling of the GPCR. Furthermore, LD probe 26 was utilized to visualize A_1_R in rat-derived neurons.

Also, Hamachi and co-workers recently presented a strategy for the development of acyl imidazole probes as tools to label receptor targets of interest.^66^ These LD probes were all based on known ligands and were conjugated to various fluorophores *via* the acyl imidazole group (Fig. 5(D)). Most interestingly, the injection of mice with the LD probes and subsequent analysis of brain homogenates and brain slices showed the receptor targets of interest could be detected, including mGlu1R, *via* the usage of probe 27.

Altogether, the above-mentioned studies show that the labelling of GPCRs on neurons and *in vivo* is feasible and not limited due to the reactivity of the respective electrophile. Besides, the activation of the receptors was not fully blocked by covalent donation of the reporter group, as agonist-induced internalization of the respective GPCR^62,67^ and calcium responses were still observed.^66^ The above-mentioned LD probes thus allowed the receptor activation and subsequent localization to be followed by microscopy techniques. Such experiments are not possible when using photo-affinity or electrophilic probes, which irreversibly block a GPCR's ligand binding pocket.

## 4. Glycan-targeting probes

**Fig. 6:**
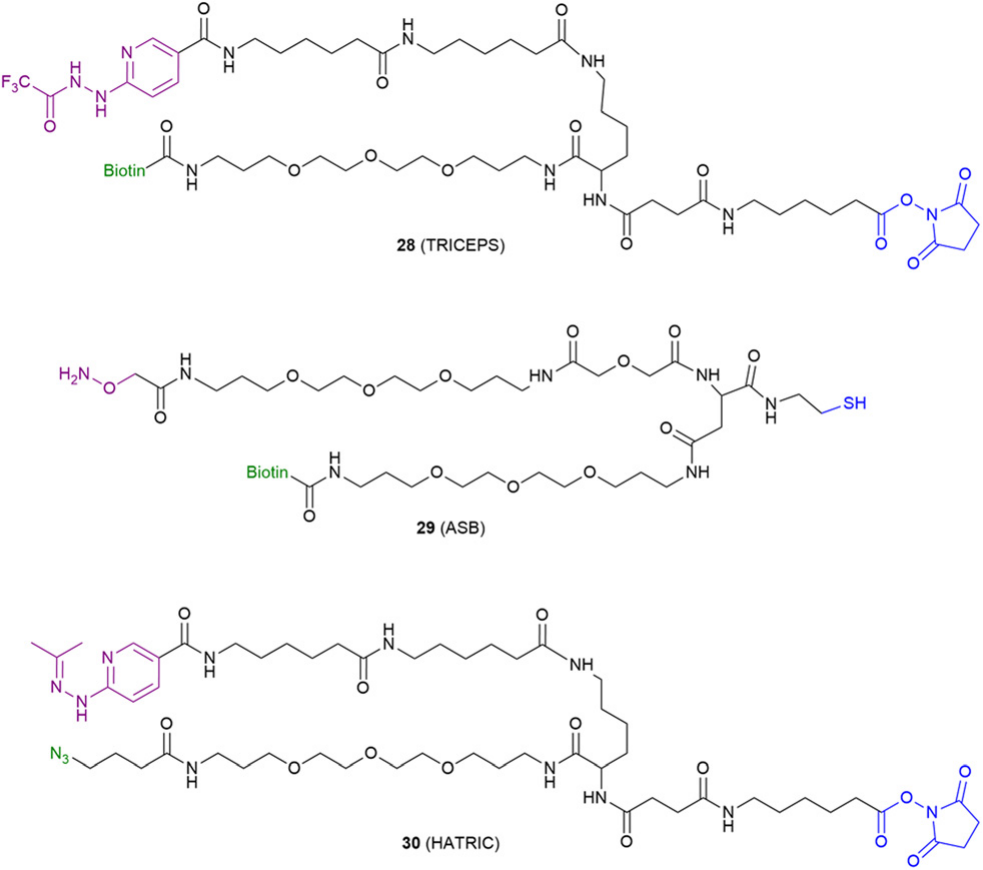
Molecular structure of glycan-targeting GPCR probes.^71–74^ Nucleophiles responsible for glycan binding are shown in purple, reporter groups are shown in green and chemical groups that allow ligand incorporation are shown in blue.

For the target identification of endogenous molecules, as well as hits from phenotypic screening, a strategy was developed that makes use of glycosylation to covalently label cell surface proteins, including GPCRs.^71,72^ First, the oligosaccharides within the glycan chain are mildly oxidized to generate aldehyde groups that function as electrophiles. Next, a trifunctional probe is added, again containing three functional moieties: (1) a high-affinity ligand; (2) a nucleophilic group; and (3) a reporter group for detection. The trifunctional probe binds to the target GPCR and subsequently forms a covalent bond with an aldehyde of a proximal glycan chain (Fig. 1(C)), allowing detection of the GPCR in biochemical assays. The first glycan-targeting probe 28 (coined trifunctional chemoproteomic reagent, or ‘TRICEPS’) utilized trifluoroacetylated hydrazine as a nucleophile, while later probes utilized aminooxy groups (‘ASB’ probe 29) and acetone-protected hydrazine groups (‘HATRIC’ probe 30) (Fig. 6).^71–74^ Notably, all the reported glycan-targeting probes needed to be ‘pre-coupled’ to a GPCR ligand prior to their utilization in biochemical assays. Pre-coupling was carried out *via* the electrophilic *N*-hydroxy succinimide ester or the nucleophilic thiol group. Either biotin or a clickable azide group was utilized as the reporter moiety of choice in these examples. Among these glycan-targeting probes, 28 was used in pull-down experiments for the detection of the apelin receptor (APLNR) in a proof of concept study,^71^ and for identification of latrophilin 2 receptor (LPHN2R) as a target for leucine-rich α-2-glycoprotein 1 (LRG1) in a target identification study.^75^

## 5. Metabolically incorporated probes

**Fig. 7:**
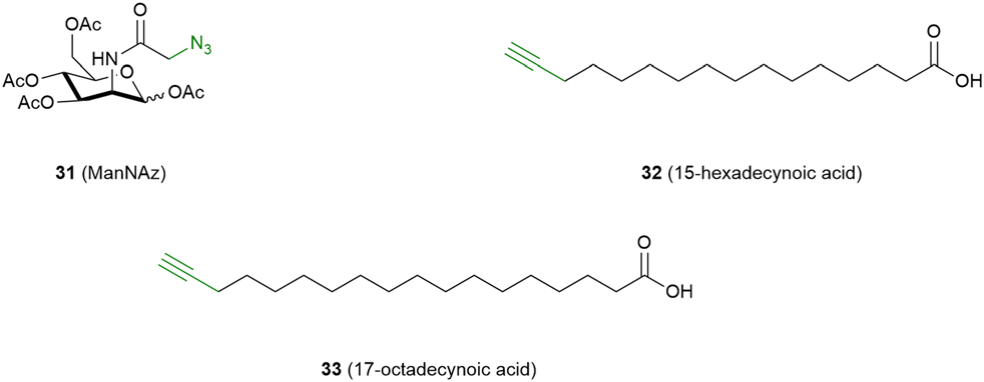
Molecular structures of probes that target GPCRs *via* metabolic incorporation.^78–83^ Alkyne groups, which function as click handles for reporter conjugation, are shown in green.

Next to targeting GPCRs *via* small molecular probes, great efforts have been made to incorporate unnatural amino acids into the peptide sequence of GPCRs, including amino acids that contain photoreactive or clickable groups.^76^ Such amino acids for genetic encoding have been extensively reviewed elsewhere and are beyond the scope of the current review.^76,77^ Nevertheless, there are two interesting strategies that incorporate small molecular probes without altering the genetic code of the GPCR (Fig. 1(D)). First, the clickable oligosaccharide 31 was metabolically incorporated in the glycan chains of proteins, among which were the histamine H3 receptor (H_3_R) (Fig. 7). Attachment of a terbium chelate *via* click chemistry and subsequent utilization of fluorescent H_3_R probes allowed detection of the H_3_R in FRET-based assays.^78^ Second, clickable variants of palmitic acids 32 and 33 were metabolically incorporated as S-palmitoyl groups. This allowed identification of the palmitoylation sites at the α_1_ adrenergic receptor (α_1_R),^79^ β_1_ and β_2_ adrenergic receptors (β_1_R and β_2_R),^80,81^ MOR,^82^ and D_2_R^83^ through SDS-PAGE and western blot experiments.

## 6. Conclusion and outlook

Over the past decade, roughly 50 small molecular probes have been developed for the covalent functionalization of GPCRs. These include affinity-based probes, ligand-directed probes, glycan-targeting probes, and metabolically incorporated probes, using either a one- or two-step labelling strategy. It should be noted that the covalent functionalization of GPCRs is not limited to these types of small molecular probes, and new types of functionalized covalent probes will most likely be developed in the future. If one had to set up new assays to label GPCRs, the electrophile and the reporter group should be carefully chosen depending on the envisioned assay setup. For example, click reagents might be avoided during live-cell experiments, while ligand-directed probes would be the tools of interest for investigating agonist-induced internalization and localization.

Thus far, most of the reported functionalized covalent probes have been used to detect the presence of the receptor in overexpressing cell lines, although in some studies, receptor expression was assessed in human blood cells^21,42^ or brain slices from rats or mice.^62,66,67^ While similar experiments could also be performed with GPCR antibodies – if available at all – a big advantage of using small molecular probes is the availability of known ligands to block the probe binding pocket prior to probe labelling, allowing for an extra positive control in the experiments. Such controls can help to rule out off-target labelling that might result from the reactive groups on the AfBPs, but could also be a problem in the case of antibodies.^10,12^ Also, covalent functionalization prevents the possible loss of reversibly bound detection moieties, allowing for a more precise detection of the cellular localization of GPCRs in live-cell experiments.^32,41,62,63,67^

Furthermore, functionalized covalent probes have been shown to be elegant tools for the target identification of bioactive molecules using pull-down proteomics.^35,49–51,75^ However, such success is not guaranteed, while the detection of GPCRs in chemical proteomic experiments can be cumbersome due to the relatively low expression levels of GPCRs, resulting in low signal-to-noise ratios.^25,34,37^ For example, in target identification studies using a photo-affinity probe based on the chemokine CXCL14, the low-density lipoprotein receptor-related protein 1 (LRP1) was detected,^84^ but not the MAS-related GPCR X2 (MRGPRX2), while the latter GPCR was found to be a target of CXCL14 in functional assays.^85^ Such differences might arise from the difficulties in measuring GPCRs in LC–MS/MS-based experiments. The careful examination of multiple variables, *e.g.* expression level, solubilization, and digestion methods, is therefore of great importance in target identification studies.^23,41,73,86,87^

Building on this, investigations have been carried out towards the detection of GPCR protein interaction partners (GPCR interactomes) by means of pull-down measurements.^23,25,30,34^ However, caution must be taken not to rule out possible off-target labelling by the respective probes and not to lose important interactions due to the use of harsh reagents and/or conditions during the sample preparation. Rigid controls should therefore be included in the experimental design of future studies that target GPCR interactomes with chemical probes.

Lastly, the covalent functionalization of GPCRs has revealed the presence of several PTMs, of which *N*-glycosylation has been reported to be the most evident.^21,32,41–43,58,63^ Glycan-targeting and clickable sugar moieties are even based on the idea of receptor glycosylation.^71–74,78^ S-Palmitoylation as a PTM has been studied with two-step metabolic fatty acid probes to investigate agonist-induced internalization,^80^ receptor stability, and trafficking for their respective GPCRs.^81,83^ Nevertheless, many questions remain regarding the location and sequence of PTMs, as well as their regulatory effects on receptor functioning.^88–90^ We can envision future studies in which metabolically incorporated probes are combined with AfBPs or ligand-directed probes in an effort to characterize all the PTMs and their effects.

In the future, the use of functionalized covalent probes will aid the thorough investigations of target GPCRs. Functionalized covalent probes could thereby allow the target identification of GPCRs as targets for currently known and unknown molecules, thereby possibly ‘de-orphanizing’ GPCRs, and allowing deciphering their roles in pathological conditions. Additionally, the covalent functionalization of target GPCRs with fluorophores will aid investigations on the lifetime and fate of GPCRs upon agonist-induced activation. Such information would be very valuable when investigating drug-induced signalling pathways, *e.g.* in the case of biased agonists. Lastly, the covalent functionalization of GPCRs with biotin, or likewise, followed by subsequent pull-down proteomics, might help to further characterize GPCRs at a molecular level, *e.g.* by analyses of the PTMs or protein interaction partners. However, in all these cases, the electrophilicity of the reactive group should be kept in mind and the appropriate controls should be added to exclude possible false hits and off-target signalling.

Altogether, there are many possibilities to make smart use of functionalized covalent probes, we would like to emphasize that these probes should not replace reversible probes, or genetic or metabolic techniques to functionalize receptors. Instead, these techniques should be complementary with one another, all yielding their own subset of information. In the future, a combined toolbox filled with reversible, covalent, genetic, and metabolic probes would be of great use in answering fundamental questions regarding GPCRs.

## Data availability

No primary research results, software or code have been included and no new data were generated or analysed as part of this review.

## Conflicts of interest

There are no conflicts to declare.
